# Nodal Yield in Level II-IV Neck Dissections in Head and Neck Squamous Cell Carcinoma

**DOI:** 10.7759/cureus.63310

**Published:** 2024-06-27

**Authors:** João V Pinto, Sofia Pedrosa, Fernando Vales, Pedro Rodrigues Pereira, Helena Silveira, Carla P Moura

**Affiliations:** 1 Otorhinolaryngology Department, Unidade Local de Saúde de São João, Porto, PRT; 2 Otorhinolaryngology Department, Faculdade de Medicina da Universidade do Porto, Porto, PRT; 3 Research, Centro de Investigação em Tecnologias e Serviços de Saúde (CINTESIS), Porto, PRT; 4 Pathology and Laboratory Medicine Department, Unidade Local de Saúde de São João, Porto, PRT; 5 Otolarhinoryngology Department, Unidade Local de Saúde de São João, Porto, PRT; 6 Genetics Department, Porto Medical School, University of Porto, Porto, PRT; 7 Research, i3S - Instituto de Investigação e Inovação em Saúde, Porto, PRT

**Keywords:** oral cavity squamous cell carcinoma, carcinoma of larynx, neck dissection, head and neck cancer surgery, head and neck cancer (hnc)

## Abstract

Objectives: The main objective of this study is to analyze factors associated with nodal yield in level II-IV selective neck dissections (NDs) and the secondary objective is to assess its impact on overall and disease-free survival.

Methods: Observational retrospective study including adult patients submitted to level II-IV ND from January 2015 to December 2021 in the otorhinolaryngology department of a tertiary hospital center.

Results: A total of 44 patients and 78 level II-IV NDs (34 bilateral and 10 unilateral) were included. The median age at diagnosis was 60 (22-74) years, and 93.2% of the patients were male. A lower nodal yield was significantly associated with previous radiotherapy (p = 0.042) and extranodal invasion (p < 0.001) and was non-significantly associated with older age (p = 0.065). Furthermore, on a *Cox* analysis adjusted to the cN status and age, the nodal yield was not associated with five-year disease-free survival (HR = 0.986; 95% CI = 0.922-1.054; p = 0.681) nor with five-year overall survival (HR = 1.006; 95% CI = 0.925-1.095; p = 0.888).

Conclusion: A reduced nodal yield in level II-IV NDs was significantly associated with previous radiotherapy and extranodal extension and non-significantly associated with age. There was no association between the nodal yield and five-year overall survival or disease-free survival.

## Introduction

Head and neck squamous cell carcinoma (HNSCC) is the sixth most prevalent cancer globally [1]. These tumors are treated, in most cases, with primary cancer resection and neck dissection (ND) [2]. NDs may be therapeutic for palpable or radiological detectable nodal metastasis or elective when there is a significant risk of occult lymph node metastasis [2]. Elective NDs are usually performed according to the patterns of drainage of the primary tumor site and the risk for each level to have occult nodal metastasis [3]. The most common indication for level I-III selective ND is oral cavity cancer, and the most frequent indications for level II-IV selective ND are oropharyngeal, hypopharyngeal, and laryngeal cancer [3].

Elective NDs are important for the pathological staging of HNSCC. In fact, the presence of nodal metastasis is associated with poorer survival, and lymph node density (number of N+ divided by nodal yield) seems to be an even stronger predictor of survival [4]. This implies that survival may be associated with a lower nodal yield and the majority of the studies are in accordance with this association [5-8], while some authors did not find an association [9,10].

The main objective of this study is to analyze factors associated with nodal yield in level II-IV selective NDs, and the secondary objective is to assess its impact on overall and disease-free survival.

## Materials and methods

An observational retrospective study including level II-IV NDs performed in adults (>18 years) with HNSCC from January 2015 to December 2021 in the otorhinolaryngology department of Hospital de São João, a tertiary hospital center in Porto, Portugal. Patients with a previous neck surgery or without sufficient data were excluded. Bilateral NDs were considered as different procedures and were reported separately.

Data collection was performed in December 2021. All demographic and clinical data were collected by analyzing patients' digital medical records, and tumor staging was in accordance with the 8th edition of the Union for International Cancer Control [11].

**Figure 1:**
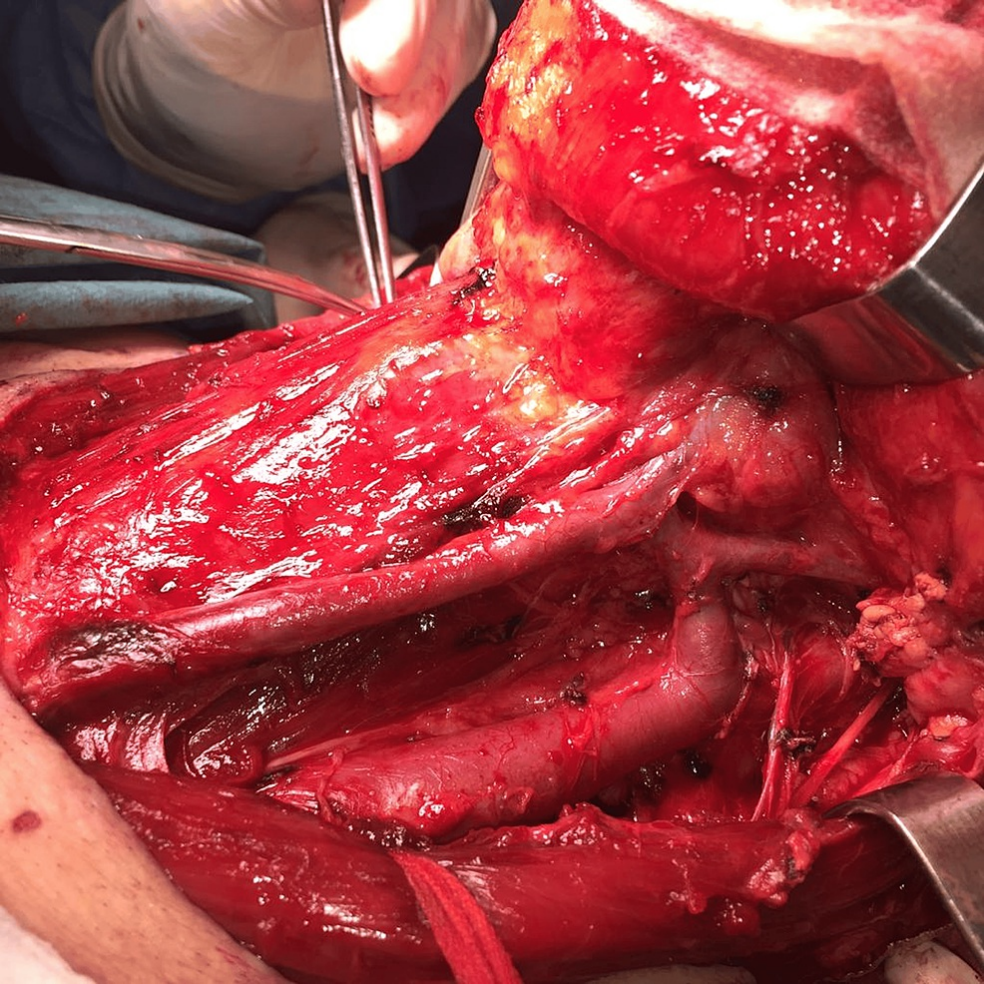
Example of a selective II-IV neck dissection (ND) before closure.

**Figure 2:**
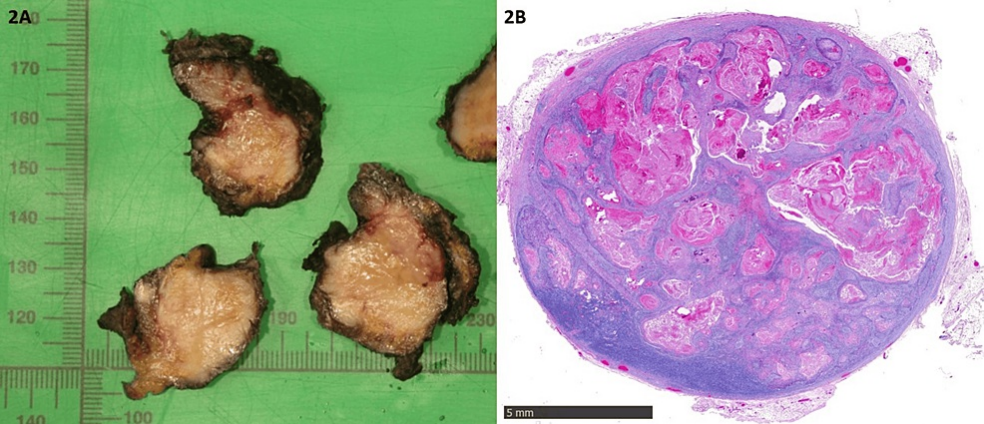
2A: Cut surface of a grossly positive lymph node with infiltrative limits sugesting extranodal extension. 2B: Microscopical examination on hematoxylin-eosin with a 20x magnification: well differentiated queratinizing squamous cell carcinoma metastasized to a lymph node.

NDs were performed by different otorhinolaryngologists experienced in head and neck surgery (Figure 1). Surgical specimens were sent for pathological examination en bloc and after adequate formaldehyde fixation were macroscopically and microscopically reported by different experienced pathologists (Figure 2). The specimens were grossly examined to isolate palpable lymph nodes, and all additional fat tissue was carefully sectioned in 1-2 mm slices to identify smaller and impalpable lymph nodes. Macroscopically negative and equivocal lymph nodes were submitted in toto, while grossly positive lymph nodes may be partially submitted for microscopic documentation of metastasis. After processing, embedding in paraffin blocks, and sectioning, the slides were stained with hematoxylin-eosin for microscopic evaluation. Reporting of lymph node status included the number of isolated and metastasized lymph nodes, greatest dimension, and presence or absence of extranodal extension.

A descriptive analysis of the patients’ characteristics was performed, taking into consideration absolute and relative frequencies for categorical variables, mean and standard deviation for normally distributed continuous variables and median and range for non-normally distributed continuous variables. The normality of continuous variables was assessed with the Kolmogorov-Smirnov test. Factors associated with nodal yield were assessed with the Mann-Whitney U test, Kruskal Wallis test, or univariate linear regression. Furthermore, five-year disease-free survival and five-year overall survival were analyzed with the Cox model, and hazard ratios (HRs) with 95% confidence interval (95% CI) were reported. To evaluate the five-year disease-free survival and five-year overall survival, we excluded patients with previous radiotherapy, previous chemoterapy, or recurrent tumors. Follow-up data from patients who were alive without disease recurrence were registered as censored at the last follow-up visit. All statistical analysis was made with the IBM SPSS Statistics for Windows, Version 27.0 (released 2020, IBM Corp., Armonk, NY), and associations were considered significant when p < 0.05.

Ethical approval was obtained from the Ethics Committee of Hospital de São João, Porto, Portugal, with reference number 27/2022.

## Results

Patients’ characteristics

A total of 44 patients and 78 level II-IV NDs (34 bilateral and 10 unilateral) were included. The patients’ demographics and clinical data are reported in Table 1. The median age at diagnosis was 60 years (range 22-74), the majority of patients were male (93.2%), and the mean body mass index was 24.19. The most frequent primary tumor location was in the larynx (n = 24; 54.6%), and the second most common was in the oropharynx (n = 6; 13.6%). Seven patients (15.9%) had previous radiotherapy and six (13.6%) had previous chemotherapy. Tumor staging is presented in Table 2.

**Table 1:** Clinical and demographic factors for the patients included in the study. BMI: body mass index; SD: standard deviation

Age; median (range)		60 (22–74)
Male gender; n (%)		41 (93.2%)
BMI; mean +/- SD		24.19 +/- 4.36
Excessive alcohol consumption; n (%)		11 (25.0%)
Tobacco consumption; n (%)		30 (68.2%)
Previous radiotherapy; n (%)		7 (15.9%)
Previous chemotherapy; n (%)		6 (13.6%)
Tumor location	Laryngeal; n (%)	24 (54.6%)
	Oropharynx; n (%)	6 (13.6%)
	Oral Cavity; n (%)	5 (11.4%)
	Hypopharynx; n (%)	3 (6.8%)
	Other; n (%)	6 (13.6%)
Follow-up in months; median (range)		20 (12-60)
Loco-regional recurrence; n (%)		9 (20.5%)
Death; n (%)		8 (18.2%)

**Table 2:** Pathological staging of the patients included in the study.

pT0	3 (6.8%)
pT1	9 (20.5%)
pT2	12 (27.3%)
pT3	6 (13.6%)
pT4	14 (31.8%)
pN0	26 (59.1%)
pN1	6 (13.6%)
pN2	6 (13.6%)
pN3	6 (13.6%

Factors associated with nodal yield

Considering the 78 NDs, the median nodal yield was 11.5 (range 0-48). Factors evaluated for an association with the nodal yield are reported on Table 3. On a univariate analysis, previous radiotherapy (p = 0.042) and extranodal extension (ENE) (p < 0.001) were significantly associated with a lower nodal yield. Furthermore, there was a non-significant decrease of total lymph nodes with older age (p = 0.065). There was no association between nodal yield and BMI (p = 0.572), pT staging (p = 0.177), pN staging (p = 0.573), leading surgeon (p = 0.096) or pathologist (p = 0.072).

**Table 3:** Univariate analysis evaluating factors associated with the nodal yield. BMI: body mass index

		Nodal yield median (range)	p
Gender	Male (n = 73)	11 (0-48)	0.771
	Female (n = 5)	17 (3-31)	
Age (correlation)		-0.052	0.065
BMI (correlation)		-0.067	0.572
Tobacco consumption	Yes (n = 54)	13 (0-48)	0.167
	No (n = 24)	10 (3-31)	
Excessive alcohol consumption	Yes (n = 20)	12.5 (1-21)	0.113
	No (n = 58)	11 (0-48)	
Previous radiotherapy	Yes (n = 11)	10 (0-20)	0.042
	No (n = 67)	12 (3-48)	
Previous chemotherapy	Yes (n = 10)	8 (0-16)	0.174
	No (n = 68)	12 (3-48)	
Extranodal extension	Yes (n = 8)	8.5 (1-26)	<0.001
	No (n = 70)	13 (0-48)	
Leading surgeon			0.096
Pathologist			0.072
pT stage			0.177
pN stage			0.573
Overall stage			0.337

Survival analysis

During a median follow-up of 20 (range 12-60) months, nine (20.5%) patients had a loco-regional recurrence and eight (18.2%) died.

To evaluate the five-year disease-free survival and five-year overall survival, we only included patients submitted to primary surgery, with no history of previous radiotherapy or chemotherapy. In a Cox model adjusted for age and cN status (cN0 or cN+), nodal yield was not associated with five-year disease-free survival (HR = 0.986; 95% CI = 0.922-1.054; p = 0.681) neither with the five-year overall survival (HR = 1.006; 95% CI = 0.925-1.095; p = 0.888).

When we have only considered patients with laryngeal cancer, the nodal yield had no association with loco-regional recurrence (HR = 0.975; 95% CI = 0.888-1.070; p = 0.595). Furthermore, considering patients with non-laryngeal cancer, the nodal yield was still not associated with disease-free survival (HR = 0.954; 95% CI = 0.836-1.088; p = 0.479).

## Discussion

We have found that a lower nodal yield in level II-IV NDs was associated with previous radiotherapy and extranodal extension and non-significantly associated with older age. Furthermore, nodal yield was not associated with five-year overall survival and five-year disease-free survival.

Nodal yield is associated with the dissected levels during ND. In fact, a previous study has reported that the mean number of nodes removed was different for each cervical nodal level and level II had a higher number of nodes removed with a mean of 9.43 ±6.78 [12]. Since NDs were sent for evaluation en bloc and levels are not marked in our standard practice, a separate evaluation for different levels was not possible. Furthermore, in opposition to other studies that have included NDs with different levels for analysis [13,14], in this study, only level II-IV selective NDs were assessed in order to limit the risk of bias. On the other hand, this has led to a smaller sample size that may have limited some associations.

The median age at diagnosis was 60 years, and there was a male preponderance as reported in other studies with NDs for HNSCC [12-15]. The majority of patients were smokers (60.8%) and 25% had a history of alcohol consumption. This is in accordance with the epidemiology of HNSCC since it is usually diagnosed in older patients with a history of tobacco and alcohol consumption [16]. Selective level II-IV NDs are usually indicated for tumors of the oropharynx, hypopharynx, or larynx [2]. Thus, the majority of the patients (75%) in this cohort had a laryngeal or pharyngeal tumor.

The median nodal yield was 11.5, and it was negatively associated with previous radiotherapy and extranodal extension. Other studies have reported a reduced nodal yield in NDs for HNSCC with previous radiotherapy [15,17,18]. Radiotherapy has also been reported to reduce nodal yield in other anatomical regions, such as in colorectal cancer [19] or esophageal cancer [20]. In fact, radiotherapy seems to lead to a reduction in the number and size of nodes [21] due to the destruction of parenchyma and replacement with fibrosis and adipose tissue [22].

We have found a non-significant association between a lower nodal yield and older age. Since advanced age was reported to decrease lymph node count in previous studies [15,23], this association may reflect the small sample size of the present study. Lymph nodes seem to have a senile involution affecting every element of the node, until it becomes translucent with the reduction of lymphoid tissue [24]. Moreover, there is also an increased fibrosis and lipomatosis with a reduction of endothelial venules [25]. Thus, Yu et al. have hypothesized that these age-related changes make it more difficult to locate lymph nodes both macroscopically and microscopically leading to a reduced nodal yield [15].

The extranodal extension was also previously associated with a lower nodal yield [13]. In the presence of extranodal extension, lymph nodes may be entangled and may be more difficult to count leading to a reduced nodal count. Although an association between BMI and total lymph node count was not found in this study, a BMI <23 was previously associated with a reduced nodal yield [13]. The authors hypothesized that malnutrition might be associated with lymphoid atrophy and altered cytokine production with a reduction in the quantity and activity of T-cells, which could lead to smaller and harder-to-distinguish lymph nodes [13]. Regarding other tumor locations, BMI did not affect the nodal yield in colorectal cancer surgery [26] but has shown an association with gastric cancer surgery [27]. Further studies are required in order to analyze the impact of BMI on the lymph node count.

Regarding the pT stage, controversy exists about its role in the total nodal lymph count. While some authors found an association and claimed a higher T stage was associated with larger lymph nodes that were easier to dissect [5,23], other studies, including the present cohort, did not find an association [12,13,28]. Furthermore, a p-16 negative status was also previously associated with a lower nodal yield in mucosal squamous cell carcinoma of the head and neck [12,13].

Many studies have aimed to find a cut-off value of minimum nodal yield for HNSCC. The American Joint Committee on Cancer (AJCC) in its 7th edition has stated that a histological examination of a radical ND should include 10 or more lymph nodes and a selective ND should include at least six nodes [29]. Ebrahim et al. concluded that selective NDs for cN0 necks in oral squamous cell carcinoma should include at least 18 nodes [30]. Moreover, Léon et al. reported a higher disease-specific mortality and regional recurrence for NDs with less than 15 dissected nodes in non-laryngeal cancers [14]. On the other hand, a standard nodal yield cut-off value is yet to be universally accepted. The median nodal yield in this study was 11.5, but this study has included patients with extranodal extension and previous radiotherapy to find factors associated with the total nodal lymph count, which has lowered overall nodal yield. Furthermore, in this study, nodal yield had no association with both the five-year overall survival and five-year disease-free survival. A review has found that nodal yield seems to correlate with disease-free survival and overall survival in non-laryngeal cancers, but not with laryngeal cancers [14]. In this study, when we separated the survival analysis in laryngeal and non-laryngeal cancers, there was still no association between nodal yield and loco-regional recurrence in both groups.

This study has some limitations. First, it is an observational study, which may be associated with a lack of data in clinical files. For example, there was no description of HPV status for some patients, which has limited its analysis. In addition to this, the sample was not large enough to conduct a multivariate analysis or to analyze survival in different tumor locations. A smaller sample size might also underestimate some associations, such as in the case of advanced age and nodal yield. Furthermore, surgeries were performed by different surgeons and pathological analysis is subjective and was performed by different pathologists, but there was no association between the leading surgeon or main pathologist with nodal yield. In the survival analysis, the median follow-up was 20 months since some patients had surgery in the two years prior to data collection. While this may limit this analysis, results were similar to what were reported in previous studies [9,10,14].

## Conclusions

A reduced nodal yield in level II-IV NDs was significantly associated with previous radiotherapy, extranodal extension, and non-significantly associated with age. There was no association between nodal yield and BMI. Furthermore, there was no association between nodal yield and disease-free survival.
